# Distribution of immune cells in head and neck cancer: CD8^+ ^T-cells and CD20^+ ^B-cells in metastatic lymph nodes are associated with favourable outcome in patients with oro- and hypopharyngeal carcinoma

**DOI:** 10.1186/1471-2407-9-292

**Published:** 2009-08-22

**Authors:** Dominik Pretscher, Luitpold V Distel, Gerhard G Grabenbauer, Michael Wittlinger, Maike Buettner, Gerald Niedobitek

**Affiliations:** 1 Department of Radiation Oncology, University Hospital Erlangen, Friedrich-Alexander-University, Erlangen-Nuremberg, Germany; 2 Institute for Pathology, University Hospital Erlangen, Friedrich-Alexander-University, Erlangen-Nuremberg, Germany; 3 Interdisciplinary Center of Clinical Research, University Hospital Erlangen, Friedrich-Alexander-University, Erlangen-Nuremberg, Germany; 4 Institutes for Pathology, Sana Klinikum Lichtenberg and Unfallkrankenhaus Berlin, Fanningerstr. 32, 10365 Berlin, Germany

## Abstract

**Background:**

Tumour infiltrating lymphocytes (TIL) are generally considered to represent a host immune response directed against tumour antigens. TIL are also increasingly recognised as possible prognostic parameters. However, the effects observed are variable indicating that results cannot be extrapolated from type of tumour to another. Moreover, it has been suggested that primary solid tumours may be ignored by the immune system and that a meaningful immune response is only mounted in regional lymph nodes.

**Methods:**

We have examined the local distribution of immune cells in tumour-related compartments in head and neck squamous cell carcinomas (HNSCC). In a second step, the prognostic impact of these cells on disease-free survival (DFS) was analysed. A total of 198 tissue cores from 33 patients were evaluated using tissue mircroarray technique and immunohistochemistry. Tumour-infiltrating immune cells were identified using antibodies specific for CD3, CD8, GranzymeB, FoxP3, CD20 and CD68 and quantified using an image analysis system.

**Results:**

We demonstrate a relative expansion of FoxP3^+ ^regulatory T-cells (Treg) and of cytotoxic T-cells among tumour infitrating T-cells. We also show that intratumoural CD20^+ ^B-cells are significantly more frequent in metastatic deposits than in primary tumours. Furthermore, we observed a reduced number of peritumoural CD8^+ ^T-cells in metastatic lymph nodes as compared to univolved regional nodes suggesting a local down-modulation of cellular immunity. All other immune cells did not show significant alterations in distribution. We did not observe an association of tumour infiltrating immune cells at the primary site with outcome. However, increased numbers of intraepithelial CD8^+ ^TIL in metastatic tumours as well as large numbers of peritumoural B-cells in lymph node metastases were associated with favourable outcome. Unexpectedly, no effect on patient outcome was observed for Treg in any compartment.

**Conclusion:**

Our results suggest that alterations in lymphocyte distribution in regional lymph nodes rather than at the primary tumour site may be relevant for patient prognosis. Moreover, we demonstrate that in addition to cellular immunity humoral immune responses may be clinically relevant in anti-tumour immunity.

## Background

Tumour-infiltrating lymphocytes (TIL) are generally thought to represent a host immune response directed against antigens expressed on tumour cells [1,2]. In particular, CD8^+ ^cytotoxic T-cells (CTL) are considered to be the major effector immune cells directed against tumour cells. This notion is supported by the observation that cytotoxic TIL are an indicator of favourable prognosis in certain carcinomas while tumour-infiltrating regulatory T-cells (Treg) have been shown to be associated with unfavourable prognosis in ovarian cancer [3-6]. Tregs have been characterised as a specialised CD4^+^CD25^+^FoxP3^+^T-cell population with the ability to inhibit the activity of effector T-cells. Although it is increasingly recognised that Treg represent a heterogeneous T-cell population which may include FoxP3^- ^cells, FoxP3 expression is still considered the most appropriate single marker for the detection of Treg in situ and most studies assessing the prognostic relevance of Treg in human cancers rest on the analysis of FoxP3^+ ^cells (e.g., Gobert et al.) [7].

Studies on TILs have largely focussed on the analysis of primary tumours. It has been suggested, however, that primary tumours located outside lymphoreticular tissues may be largely ignored by the immune system and that development of a tumour-specific immune response requires entry of tumour cells into secondary lymphoid tissues [8]. Recent data suggest that changes in lymphocyte distribution may occur in regional tumour-draining lymph nodes and may play an important role for prognosis of cancer patients. For example, in patients with prostatic cancer the immune profile of regional cancer draining lymph nodes was altered as compared to uninvolved draining lymph nodes and control nodes obtained from other sites. Notably, regional metastatic and uninvolved nodes contained less CD20^+ ^B-cells and more CD8^+ ^T-cells than control nodes [9]. In gastric carcinomas an elevated number of Tregs was detected in tumour draining lymph nodes compared to uninvolved mesenteric nodes [10]. In early stage breast cancer, sentinel and axillary lymph nodes displayed reduced numbers of CD4^+ ^and CD8^+ ^T-cells as compared to control lymph nodes while highest numbers of CD1a^+ ^dendritic cells were seen in uninvolved axillary nodes [11]. Some of these effects, notably a reduction in the numbers of CD4^+ ^T-cells were observed in draining axillary nodes even in the absence of metastatic deposits suggesting that alterations of immune profiles in regional lymph nodes may occur independently of tumour invasion [11]. In that study, large numbers of CD4^+^-lymphocytes and of CD1a^+^-dendritic cells predicted more precisely for NED-survival than axillary lymph node metastasis per se indicating an important functional role of an immune cell distribution shift [11].

Squamous cell carcinomas of the head and neck region (HNSCC) represent a group of tumours occuring at various sites including the oral mucosa and the palatine tonsils. Adding to this diversity is the recent observation that a proportion of these cancers, notably tonsillar carcinomas, are associated with human papillomavirus (HPV) infection [12]. High density of lymphocytic infiltration has been identified as favourable specific marker in HNSCC [13]. Specifically, large numbers of CD3^+ ^T-cells were associated with favourable outcome in several studies [14,15]. A specific effect of CD8^+ ^CTL is controversial [16,17] while a reduction of CD8^+ ^lymphocytes in regional lymph nodes from HNSCC patients has been reported [18]. Unexpectedly in one study, FoxP3^+ ^Treg were associated with improved outcome in HNSCC [16].

Thus, results observed in different cancer models are variable and a clear picture as to the role of TIL has not yet emerged. It appears, that any analysis of the prognostic impact of TIL in solid tumours cannot simply rely on the study of primary tumour samples but has to take other factors into account such as composition of TIL subsets and distribution in different anatomical sites related to the primary tumour. In view of the limited evidence available in this respect for head and neck squamous cell carcinoma (HNSCC), the aim of this study was to evaluate further the morphological distribution and prognostic impact of regulatory T-cells (FoxP3^+^), various T-cell subsets (CD3^+^, CD8^+^, GranzymeB^+^), and macrophages (CD68^+^) in four different tumour-related compartments, i.e. primary tumour, lymph node metastases, peritumoural area in metastatic lymph nodes and uninvolved draining lymph nodes of head and neck cancer patients. Since studies of HNSCC and other cancer have suggested a beneficial effect of B-cells on outcome [19-21], we also included B-cells (CD20^+^) in our analysis.

## Methods

### Patient Selection

Between 1998 and 2004, a total of 33 patients with histologically proven squamous cell carcinoma of the oro- and hypopharynx were included in this study. This study was approved by the ethics committee of the Friedrich-Alexander-University and informed consent was obtained from the patients involved. All studies were done on tissue samples taken for diagnostic or therapeutic purposes and filed in the archives of the Institute for Pathology, University Hospital Erlangen. Tissues were not collected specifically for the purpose of this study. Treatment consisted of resection of primary tumour as well as a neck dissection followed by radiotherapy with (n = 17) and without chemotherapy (n = 16). Patients were treated in a randomized phase III-trial of postoperative radiation vs. radiochemotherapy. Details of the protocol are given elsewhere [22].

Briefly, inclusion criteria for enrolment in this study were as follows:

- squamous cell carcinoma

- primary treatment by radical surgery

- T category: pT4 or pT3 R1 or

- N category:

○ ≥ 3 pathologically involved lymph nodes or

○ extracapsular growth of metastasis in N+ nodes or

○ lymphangiosis carcinomatosa

All patients were initially assessed by a multidisciplinary team of head and neck surgeons, radiation oncologists, medical oncologists and radiologists. Staging procedures included chest X-ray, liver ultrasound and upper gastrointestinal endoscopy as well as tracheoscopy. Both neck sides and the supraclavicular region were assessed clinically and by ultrasound as well as by histopathological examination after surgery and neck dissection. Detailed patient characteristics are displayed in table 1.

**Table 1:** Patient characteristics

		n	(%)
**All patients**		33	100

**Gender**	Male	32	(97)

	Female	1	(3)

**Age (median, 53 years)**			

	36–40	1	(3)

	41–50	10	(30)

	51–60	15	(46)

	61–70	7	(21)

**Tumor site**	Tonsils	8	(24)

	Oropharynx	12	(36)

	Hypopharynx	5	(16)

	Mouth	8	(24)

**pTN category**	T1 N2	7	(21)

	T1 N3	1	(3)

	T2 N0	1	(3)

	T2 N1	2	(6)

	T2 N2	5	(16)

	T3 N1	1	(3)

	T3 N2	5	(16)

	T4 N0	1	(3)

	T4 N1	1	(3)

	T4 N2	6	(19)

	TX N1	1	(3)

	TX N2	2	(6)

**AJCC Stage**	I	0	(0)

	II	1	(3)

	III	4	(12)

	IV	28	(84)

**p16 Status**	positive	10	(31)

	negative	22	(69)

### Tissue miroarrays and evaluation of tumour infiltrating lymphocytes

Paraffin-embedded samples of the primary tumour as well as of involved and uninvolved lymph nodes available from 33 patients with oro- and hypopharyngeal squamous cell carcinoma were processed into tissue microarrays (BioCat, Heidelberg, Germany) using a core diameter of 2 mm. Six cores were taken per patient, two cores from the primary tumour, 2 cores from metastatic deposits in lymph nodes, 1 core from peritumoural lymphatic tissue of metastatic (N+) nodes and one core from lymph nodes without evidence of metastasis, resulting in a total of 198 cores. Immunohistochemistry of paraffin sections was carried out using a standard streptavidin biotinylated alkaline phosphatase (ABC-AP, DakoCytomation, Hamburg, Germany) method or tyramide signal amplification followed by ABC-AP (only for FoxP3). The following antibodies were used: CD3, CD8, CD20, CD68 and Granzyme B (all DakoCytomation, Hamburg, Germany) and FoxP3 (mouse monoclonal, clone 236A/E7, abcam, Cambridge, UK). p16-specific immunohistochemistry was carried out using a commercially available kit (mtm laboratories, Heidelberg, Germany). Cases were scored positive if the majority of cells demonstrated strong nuclear staining or negative if no staining was seen or only a small percentage of tumour cells was labelled. The results are included in table 1.

By use of a standard light microscope, images were acquired with a CCD-camera using a 20× objective, transferred to a PC and semiautomatically evaluated using the image analysis software COUNT (Biomas, Erlangen, Germany). For quantification purposes, mean numbers of lymphocytes following the analysis of 3–6 images from primary and metastatic tumour were taken, respectively. Specimens of uninvolved draining lymph nodes and peritumoural lymphatic tissue from N+ nodes were analyzed by quantification of 2 images, respectively. Numbers of labeled tumour-infiltrating cells in primary tumour and metastatic deposits in N+ nodes were determined using an image analysis system (Biomas, Erlangen, Germany) in relation to 100 tumour cells (Labeling index, LI) as described previously, counting only lymphocytes admixed with epithelial tumour cells. In uninvolved lymph nodes and in peritumoural lymphatic tissue of N+ nodes, the number of lymphocytes was quantified per area of one image (i.e. 0.087 mm^2^).

The quantitative results for the infiltration of each of these compartments are displayed in table 2.

**Table 2:** Median, range and mean labeling index for different immune cells (CD3+, CD8+, CD20+, CD68+, FoxP3+, Granzyme B+) within primary tumor, metastatic lymph nodes and uninvolved regional lymph nodes

TIL		Primary- tumor	Intraepithelial N+ node	Peritumoral N+ node	Non-metastatic node
		cells per 100 tumor cells		cells per image (0.087 mm^2^)	

**CD3**					
	
	Median (range)	**3.04** (0–19)	**3.52** (0.16–13.52)	**204.5** (60–341)	**183** (54–348)
	
	Mean ± SE	**4.36 ± 4.92**	**4.34 ± 4.64**	**209.1 ± 68.5**	**183.4 ± 69.3**

**CD8**					
	
	Median (range)	**2.49** (0.19–17.79)	**1.29** (0.16–16.23)	**37.5** (10–149)	**88** (25–308)
	
	Mean ± SE	**3.54 ± 4.48**	**3.31 ± 4.71**	**45.8 ± 37.5**	**108.5 ± 69.8**

**CD20**					
	
	Median (range)	**0.23** (0.11–5.26)	**1.76** (0.15–5.22)	**192** (19–631)	**148.5** (14–321)
	
	Mean ± SE	**0.91 ± 1.48**	**2.53 ± 2.93**	**200.6 ± 124.3**	**148.9 ± 83.9**

**CD68**					
	
	Median (range)	**1.02** (0.15–5.22)	**1.05** (0–4.93)	**5.5** (1–17)	**4** (1–23)
	
	Mean ± SE	**1.17 ± 1.26**	**1.37 ± 1.26**	**6.5 ± 4.7**	**7 ± 6.2**

**FoxP3**					
	
	Median (range)	**1.81** (0.15–13.0)	**2.26** (0.32–18.4)	**44** (4–134)	**49.5** (10–139)
	
	Mean ± SE	**2.88 ± 2.90**	**2.96 ± 3.29**	**49.0 ± 33.2**	***53.6 ± 26.4***

**Granzyme B**					

	Median (range)	**1** (0–21)	**0** (0–10)	**0** (0–54)	**3** (0–39)
	
	Mean ± SE	**2.03 ± 3.93**	**1.39 ± 2.70**	**4.79 ± 11.52**	**5.27 ± 7.96**

### Statistical analysis

Disease-free survival (DFS) was the time from initial histological diagnosis until last follow-up or the appearance of local, nodal or distant recurrence. The correlation between the infiltration of TILs and other leukocytes in different compartments was assessed using Pearson correlation procedure. All statistical analyses were performed with the SPSS for Windows software (Version 15.0). DFS rates were generated according to the Kaplan-Meier method. Comparison of survival rates was performed by use of the log rank test using median numbers as cut-off, no multivariate analyses were performed due to small number of events.

## Results

### Patterns of TIL-infiltration in different compartments

**Figure 1:**
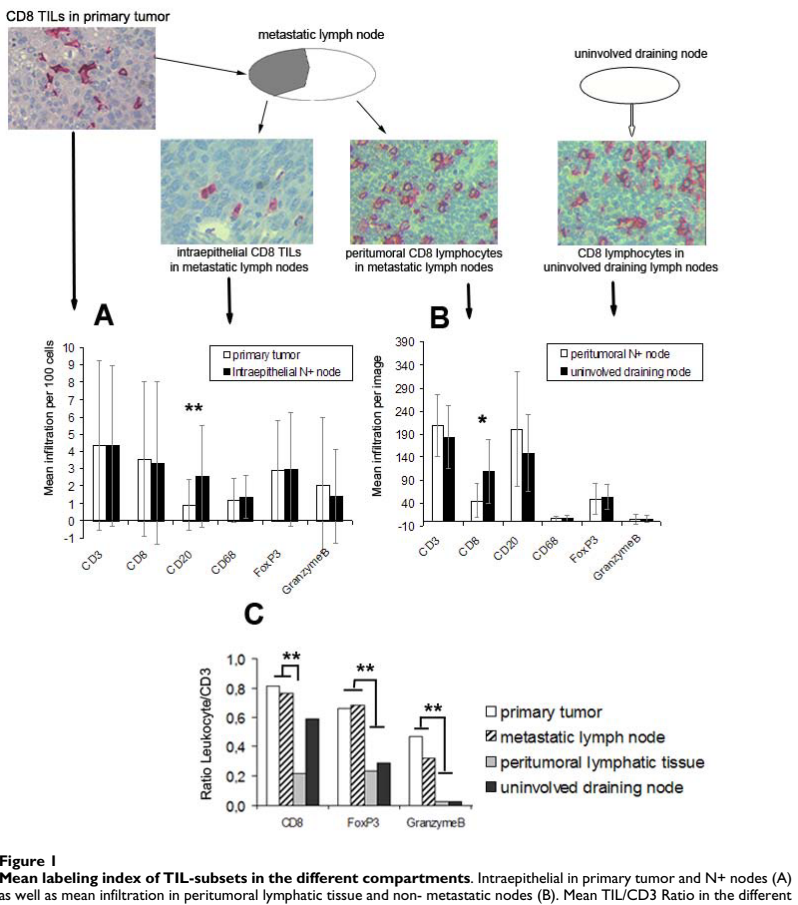
**Mean labeling index of TIL-subsets in the different compartments**. Intraepithelial in primary tumor and N+ nodes (A) as well as mean infiltration in peritumoral lymphatic tissue and non- metastatic nodes (B). Mean TIL/CD3 Ratio in the different compartments (C). * p < 0.05, ** p < 0.01.

Infiltration of lymphocytes and macrophages was studied in the following four compartments: (i) primary tumour (ii) metastatic tumour,(iii) peritumoural areas in metastatic lymph nodes, and (iv) uninvolved draining lymph nodes (Figure 1). In primary tumour and metastatic tumour in regional lymph nodes labeling index, i.e., number of intraepithelial cells per 100 tumour cells, was used to quantify TIL whereas in peritumoural lymphatic tissue and in uninvolved lymph nodes numbers of lymphocytes per area (0.087 mm^2^) were determined. Overall the numbers of TIL in primary and metastatic tumour were similar, and this was also true for the comparison of peritumoural lymphoreticular tissue in metastatic lymph nodes and uninvolved lymph nodes (Figure 1a, b, Table 2). However, there were two notable exceptions. Mean CD20^+ ^cell infiltration was significantly increased in metastatic lymph node deposits compared to primary tumour (p < 0.005) and CD8^+ ^lymphocytes were significantly more frequent in uninvolved draining nodes compared to peritumoural lymphatic tissue of metastatic lymph nodes (p < 0.022). FoxP3^+ ^Treg were similarly frequent in both tumourous compartments. The ratio of TIL subsets/CD3^+ ^cells was used to compare the infiltration in the four different compartments. Using this approach, we show that, as a proportion of CD3^+ ^T-cells, tumour infiltrating CD8^+ ^and Granzyme B^+ ^cytotoxic T-cells as well as FoxP3^+ ^Treg were relatively enriched in primary and metastatic tumours when compared to uninvolved lymphoid tissue (Figure 1c).

### Prognostic impact of TIL in different tumour compartments

As in our previous study [23] the median was used as a cut-off to separate patient groups with large and small numbers of tumour-infiltrating immune cells.

**Figure 2:**
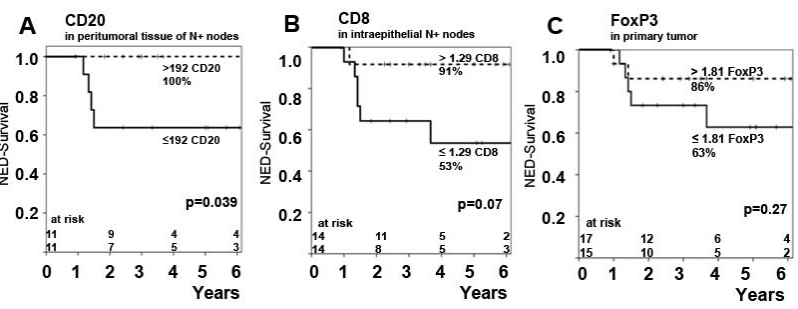
**Impact of different TIL subgroups in various compartments on NED-survival rates**. Impact of CD20^+ ^– lymphocyte infiltration of peritumoral lymphatic tissue(A), impact of intraepithelial CD8^+^-TIL in N+ nodes (B) and impact of FoxP3^+^-TIL infiltration of primary tumor (C).

Patients with high numbers of peritumoural infiltration of CD20^+ ^B-lymphocytes in metastatic lymph nodes had a significantly better outcome: DFS rates at 5 years were 100% vs. 64% for patients with a high versus low peritumoural accumulation (>192 CD20^+ ^cells vs. <192 CD20^+ ^per 0.087 mm^2^, p = 0.04) (figure 2A).

An increased intraepithelial infiltration of CD8^+^-TIL (>1.29 CD8^+ ^TIL LI) in metastatic lymph nodes was associated with a superior DFS rate of 91% vs. 53% (p = 0.07) (Figure 2B).

There was a trend towards improved DFS rates for patients with an increased infiltration of FoxP3^+^-TIL in the primary tumour (Figure 2C) with 86% vs. 63% (p = 0.27) as well as FoxP3^+^-TIL in N+ nodes with 84% vs. 53% (p = 0.17). A similar though again statistically not significant result was found for the impact of peritumoural FoxP3^+ ^lymphocytes in metastatic lymph nodes: DFS rates at 5 years were 91% vs. 62% (p = 0.18) (table 3).

**Table 3:** Impact of infiltration by various immune cell subtypes on disease-free survival (DFS)

	Cut-off value*	DFS	p
**Immune cells in primary tumor**			

CD3	>3.04 (n = 16) vs. ≤ 3.04 (n = 16)	85% vs. 67%	0.4

CD8	>2.49(n = 16) vs. ≤ 2.49(n = 16)	79% vs. 69%	0.29

FoxP3	>1.81 (n = 17) vs. ≤ 1.81(n = 15)	86% vs. 63%	0.27

CD20	>0.23 (n = 18) vs. ≤ 0.23(n = 13)	83% vs. 81%	0.8

CD68	>1.02 (n = 16) vs. ≤ 1.02(n = 16)	73% vs. 75%	0.8

Granzyme B	>0 (n = 18) vs. = 0(n = 15)	82% vs. 68%	0.53

**Intraepithelial immune cells in metastatic lymph nodes**			

CD3	>3.52 (n = 13) vs. ≤ 3.52 (n = 14)	77% vs. 62%	0.26

CD8	>1.29 (n = 14) vs. ≤ 1.29 (n = 14)	91% vs. 53%	***0.07***

FoxP3	>2.26 (n = 14) vs. ≤ 2.26 (n = 13)	84% vs. 53%	0.17

CD20	>1.76 (n = 12) vs. ≤ 1.76 (n = 11)	82% vs. 60%	0.29

CD68	>1.05 (n = 14) vs. ≤ 1.05 (n = 13)	67% vs.73%	0.9

Granzyme B	>0 (n = 11) vs. = 0(n = 19)	80% vs. 68%	0.46

**Peritumoral immune cells in lymphatic tissue of metastatic nodes**			

CD3	>204.5 (n = 13) vs. ≤ 204.5 (n = 13)	66.7% vs. 80.8%	0.25

CD8	> 37.5 (n = 12) vs. ≤ 37.5 (n = 12)	72.7% vs. 76.4%	0.53

FoxP3	> 44 (n = 11) vs. ≤ 44 (n = 12)	90.9% vs. 62.3%	0.18

CD20	> 192 (n = 11) vs. ≤ 192 (n = 11)	100% vs. 63.6%	***0.039***

CD68	> 5.5 (n = 13) vs. ≤ 5.5 (n = 13)	76.9% vs. 81.5%	0.5

Granzyme B	>0 (n = 15) vs. = 0 (n = 15)	78.6% vs.67.3%	0.65

**Immune cells in uninvolved regional lymph nodes**			

CD3	>183 (n = 15) vs. ≤ 183 (n = 16)	75.0% vs. 73.3%	0.675

CD8	>88 (n = 15) vs. ≤ 88 (n = 16)	84.6% vs. 65.6%	0.337

FoxP3	>49 (n = 15) vs. ≤ 49 (n = 16)	78.6% vs. 71.1%	0.905

CD20	>147 (n = 15) vs. ≤ 147 (n = 16)	79% vs. 68.6%	0.779

CD68	>4 (n = 12) vs. ≤ 4 (n = 17)	75.8% vs. 68.8%	0.424

Granzyme B	>0 (n = 13) vs. = 0 (n = 15)	73.3% vs.68.8%	0.939

*Median was used as cut-off

The infiltration of the primary tumour by CD3^+^, CD8^+^, CD20^+^, CD68^+ ^and Granzyme B^+ ^had only limited influence on DFS (Table 3). Similarly, an analysis of the ratios of leukocyte subsets over FoxP3^+^-TIL did not reveal any influence on prognosis (not shown).

## Discussion

Tumour infiltrating lymphocytes are generally considered to represent host immunity against tumours [1,2]. Evidence to support this notion mainly comes from clinical studies showing that in certain tumour models, large numbers of CD8^+ ^TIL are associated with a favourable outcome [3-6]. However, this is not applicable to all tumour models. Thus, cytotoxic TILs were found to have a negative effect on patients' prognosis in anal cancer or Hodgkin lymphoma [23,24]. The discovery of Treg has further complicated this issue. As anticipated, tumour infiltrating FoxP3^+ ^Treg have been reported to be negative prognostic factors for ovarian, esophageal and hepatocellular cancer [25-27]. Again, however, other studies have demonstrated different effects. Thus, FoxP3^+ ^Tregs were associated with favourable prognosis in patients with Hodgkin or follicular lymphoma as well as in HNSCC patients [16,24,28], while no effect was reported for anal carcinomas [23]. Moreover, it has been suggested that absolute numbers may be less relevant than TIL subset ratios. For example, low numbers of Treg together with large numbers of cytotoxic T-cells have been demonstrated to indicate favourable outcome in ovarian cancer while the same constellation is indicative of poor prognosis in Hodgkin lymphoma [6,24].

In addition to these issues, a few studies have begun to address the question of heterogenity of immunological microenvironments in primary carcinoma, lymph node metastases and uninvolved regional lymph nodes. This is important since it has been suggested that many solid cancers may be invisible to the immune system at the primary site and are only recognised following metastasis into regional lymph nodes [8,29].

HNSCC represent a heterogenous group of tumours characterised by squamous differentiation and includes tumours arising at diverse anatomical sites such as oral mucosa and palatine tonsils. Our study was restricted to carcinomas arising in the oro- and hypopharynx in order to minimise site-related variability. It has become clear in recent years, that a substantial proportion of HNSCC are associated with human papillomavirus (HPV). Overall, up to 20% of HNSCC are HPV-positive [12]. However, the rate of positivity varies depending on site with highest numbers of HPV-positive cases being observed in the oropharynx [12]. We used p16 expression as a surrogate marker of HPV infection. p16 expression was found in 31% of cases, in keeping with the high proportion of oropharyngeal carcinomas in our series. It has been shown recently, that HPV-associated HNSCC differ from the HPV-negative cases with regards to risk factors and notably are not associated with smoking [30]. It is likely, that HPV status of tumour cells and concomitant expression of viral proteins will affect the nature of local immune reactions. This will have to be addressed in future studies.

We have investigated the distribution of tumour infiltrating leukocytes in tumour-related compartments of HNSCC. We firstly show that, as a proportion of CD3-positive T-cells, the composition of T-cell subsets within primary and metastatic carcinomas varies from that seen in adjacent lymph node tissue. Notably, we confirm previous studies reporting an increase in the proportion of FoxP3-positive Treg and of cytotoxic T-cells among TILs [2]. This observation supports the notion that tumour cells actively modulate their microenvironment, possibly by the expression of cytokines and chemokines.

We also show that the numbers of tumour infiltrating CD3^+ ^T-cells, CD8^+ ^and GranzymeB^+ ^cytotoxic T-cells as well as of FoxP3^+ ^Treg are similar in primary HNSCC and in lymph node metastases. A notable exception were CD20^+ ^intratumoural B-cells which were significantly more frequent in metastatic deposits than in primary tumours. While we cannot prove that the immunoglobulins expressed by these B-cells are specific for antigens expressed in the tumours, this observation is in keeping with the notion that a relevant interaction between tumours and the humoral immune system occurs in lymph nodes rather than at the primary tumour site.

Furthermore, a significant reduction of CD8^+^-lymphocytes in peritumoural areas in metastatic lymph nodes as compared to uninvolved regional lymph nodes was observed. This finding is in agreement with previous studies of head and neck as well as breast cancers and suggests a general suppression of cytotoxic T cell immunity in the immediate vicinity of the neoplastic cells [11,31,32]. It has been suggested that a cytokine-induced local shift towards a Th2-predominant pattern of immunity may enable tumours to avoid host immunity [33], and our observation is in line with this notion.

To assess the significance of the differential accumulation of TIL subsets, we examined the effect on DFS in our patients. Large numbers of intraepithelial CD8^+ ^TIL tended to be associated with a favourable outcome. This effect was stronger for metastatic lymph node deposits than for primary tumours, although neither reached statistical significance, probably due to the relative small number of cases. Nevertheless, this observation is well in line with previous studies of other solid malignancies [3-6].

Patients with large numbers of CD20^+ ^B-cells generally also showed favourable outcome. There were however, clear differences when analysing different compartments. No effect was seen in primary tumours, while a statistically significant improvement of DFS was noted in those patients showing large numbers of CD20^+ ^cells in the peritumoural lymphoid tissue in metastatic lymph nodes. Again, this observation underlines the potentially important role of B-cells in anti-tumour immunity.

All other leukocyte subsets, including FoxP3^+ ^Treg, did not show any significant influence on outcome. This was unexpected since several previous studies have suggested an adverse prognostic effect of Treg in ovarian carcinoma and HNSCC [25]. However, in other malignancies opposite effects have been observed, and our previous study of anal carcinomas has not shown a significant prognostic influence of Treg [23]. Thus, the role of Treg in anti-tumour immunity is likely to be heterogeneous. In addition, it is becoming increasingly clear that FoxP3 expression is not restricted to Treg. Indeed, FoxP3 expression has been demonstrated in normal and neoplastic epithelial cells. Since in our cases the tumour cells were consistently FoxP3-negative, this did not interfere with the interpretation of our results. In addition, regulatory functions in tumour immunity may not be restricted to FoxP3-positive Treg and includes certain myeloid-derived cells [2]. Thus, a comprehensive analysis of the clinical impact of immune cells with regulatory function in HNSCC will have to include cell populations in addition to Treg as defined by FoxP3 expression.

## Conclusion

In conclusion, we show that patterns of tumour-related leukocyte infiltration vary between primary tumours and metastatic lymph nodes in head and neck cancers with a local decrease in the number of CD8^+ ^T-cells and an increase of CD20^+ ^B-cells being the most relevant findings. The notion that a suppression of local cellular immunity may be a strategy by which tumours may evade host immunity [33] is supported by our observation that patients with large numbers of intraepithelial CD8^+ ^cells in metastatic tumour deposits showed a more favourable outcome. Unxpectedly, we also found a beneficial effect of large numbers of CD20-positive B-cells in metastatic lymph nodes suggesting an important role for humoral anti-tumour immunity.

## Abbreviations

HNSCC: Head and neck squamous cell carcinoma; DFS: Disease free survival; TIL: Tumour infiltrating lymphocytes; Treg: Regulatory T cells.

## Competing interests

The authors declare that they have no competing interests.

## Authors' contributions

The study concept was designed by GN and GG, the experimental studies were accomlished by DP and LD. Data acquistion by DP, MW and MB. The Manuscript was edited by DP, LD, GN and GG. All authors have read and approved the manuscript.

## Pre-publication history

The pre-publication history for this paper can be accessed here:

http://www.biomedcentral.com/1471-2407/9/292/prepub
